# A simple and accurate SNP scoring strategy based on typeIIS restriction endonuclease cleavage and matrix-assisted laser desorption/ionization mass spectrometry

**DOI:** 10.1186/1471-2164-9-276

**Published:** 2008-06-09

**Authors:** Sun Pyo Hong, Seung Il Ji, Hwanseok Rhee, Soo Kyeong Shin, Sun Young Hwang, Seung Hwan Lee, Soong Deok Lee, Heung-Bum Oh, Wangdon Yoo, Soo-Ok Kim

**Affiliations:** 1 Research & Development Center, GeneMatrix, Inc., Yongin, 446-913, South Korea; 2 DNA Analysis Lab, Supreme Public Prosecutor's Office, Seoul 137-730, South Korea; 3 Department of Forensic Medicine, Seoul National University College of Medicine, 28, Yongon-Dong, Chongno-Gu, Seoul, 110-799, South Korea; 4 Department of Laboratory Medicine, University of Ulsan College of Medicine and Asan Medical Center, Seoul, 138-736, South Korea

## Abstract

**Background:**

We describe the development of a novel matrix-assisted laser desorption ionization time-of-flight (MALDI-TOF)-based single nucleotide polymorphism (SNP) scoring strategy, termed Restriction Fragment Mass Polymorphism (RFMP) that is suitable for genotyping variations in a simple, accurate, and high-throughput manner. The assay is based on polymerase chain reaction (PCR) amplification and mass measurement of oligonucleotides containing a polymorphic base, to which a typeIIS restriction endonuclease recognition was introduced by PCR amplification. Enzymatic cleavage of the products leads to excision of oligonucleotide fragments representing base variation of the polymorphic site whose masses were determined by MALDI-TOF MS.

**Results:**

The assay represents an improvement over previous methods because it relies on the direct mass determination of PCR products rather than on an indirect analysis, where a base-extended or fluorescent report tag is interpreted. The RFMP strategy is simple and straightforward, requiring one restriction digestion reaction following target amplification in a single vessel. With this technology, genotypes are generated with a high call rate (99.6%) and high accuracy (99.8%) as determined by independent sequencing.

**Conclusion:**

The simplicity, accuracy and amenability to high-throughput screening analysis should make the RFMP assay suitable for large-scale genotype association study as well as clinical genotyping in laboratories.

## Background

Genetic differences contributed to phenotypic diversity of humans or pathogens, including variation in disease susceptibility and drug response. The genotypic analysis to identify the polymorphisms that differentiate one individual or strain from another has become increasingly important as a prognostic measure of disease courses and to enable choice of more efficacious therapeutic or preventive options based on individual genetic makeup. Due to the complexity of many common, chronic diseases and quantitative traits and the confounding effects of disease heterogeneity, gene-gene interaction, and gene-environment interaction, a large number of the polymorphisms must be surveyed in numerous individuals. These progresses highlight the need for rapid, accurate, and efficient methods that permit high throughput genotyping.

The most commonly used methods for genotype readout are based either on fluorescence or mass spectrometry (MS). Fluorescence readout is quite sensitive but often relies on secondary reporter systems for detection [1,2]. In contrast, MS readout has the advantage of directly detecting fragments containing the original DNA sequence information and thereby potentially reduces false positive and false negative results [3]. Even though MS did not contribute to the human genome-sequencing project, it has become an essential tool in both protein and DNA analyses in the past decade, as well as the key technology in the emerging fields of proteomics and functional genomics [4]. Developed in the late 1980s, MALDI-TOF provided fast and accurate measurements of the molecular masses of short DNA sequences [5,6]. The ability to measure directly the mass-to-charge (m/z) ratio of biomolecules with high accuracy made a wide range of bio-analytical applications available to MS analysis [7-9]. Because of its speed, accuracy, and sensitivity, MALDI-TOF MS has become a powerful tool for the efficient sequencing of short DNA fragments as well as genotyping of single nucleotide polymorphisms (SNPs) [10-16]. In addition, the strength of MS lies in the fact that it uses an intrinsic property of molecules, their masses. MS directly assesses the nature of the PCR products, whereas other technologies only indirectly measure PCR products, either through hybridization or by sequencing reactions, which use PCR products as templates. Procedures have been widely used that use PCR products as templates to which oligonucleotide primers are hybridized, base-extended and then analyzed by mass spectrometry. These assays can be useful, but they fail to employ one of key advantages of mass spectrometry that the analysis of PCR products can be direct. Genotyping by creating or abolishing recognition sites for restriction enzymes, similar to conventional restriction fragment length polymorphism (RFLP) analysis, has been used in combination with MALDI mass spectrometric detection [17]. Either naturally occurring restriction sites were used or base changes were incorporated in one of the PCR primers to give a recognition site with one of the alleles of the polymorphic site. However, only a small number of polymorphisms will alter known restriction sites, and the design of amplification primers to create restriction sites in connection with one allele is not straightforward in most cases, reducing the usefulness of this approach to very special circumstances.

Here, we describe the development of a novel MALDI-TOF MS-based SNP scoring strategy, termed RFMP, that is suitable for genotyping SNPs in a simple, efficient, and high-throughput manner. The assay is based on PCR amplification and mass measurement of oligonucleotides containing a polymorphic base, to which a typeIIS restriction endonuclease recognition was introduced by PCR amplification. We demonstrated fast, reliable genotyping of five SNP markers in methylenetetrahydrofolate reductase (*MTHFR*) gene, known to be associated with hyperhomocysteinemia and cardiovascular diseases, using RFMP assay and also assessed the potential for application to determination of allele frequencies in DNA pools.

## Results and Discussion

### RFMP strategy for SNP scoring

**Figure 1:**
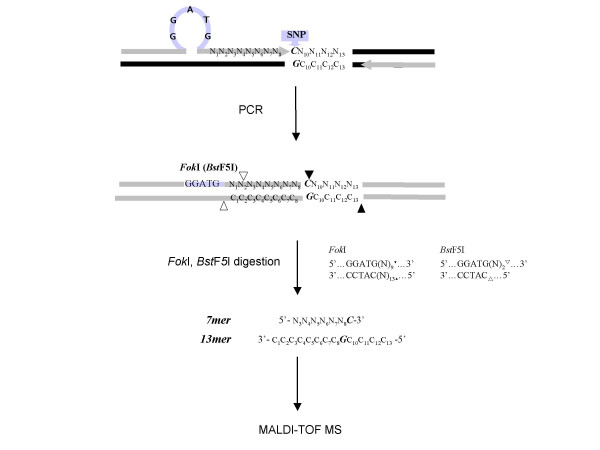
**Schematic summary of the RFMP genotyping strategy**. PCR was done with primers designed to introduce a typeIIS restriction endonuclease recognition sequence (*Fok*I = *Bst*F5I; GGATG) 9 bases ahead of the polymorphism site. The enzymatic cleavage of the products leads to excision of two oligonucleotide fragments (7 mer and 13 mer) containing the variation site, and then masses of the resulting oligonucleotide fragments were examined by MALDI-TOF MS. Cleavage sites of *Fok*I and *BstF*5I, an isoschizomer for *Fok*I, are indicated by filled and blank triangles, respectively, and restriction endonuclease recognition site and primers by shaded bar and shaded arrows, respectively. One-base gap replaced by the artificial sequences and potential SNP site are denoted by blank space and a bold italic letter, respectively.

The RFMP assay is based on mass spectrometric analysis of small DNA fragments containing site of polymorphism, as illustrated in Fig. 1. The first step requires PCR amplification using primers flanking the altered bases. The forward primer was designed to introduce a *Fok*I site (an isoschizomer of *Bst*F5I) in the amplified product by substituting the restriction recognition sequence GGATG for the ninth base upstream of the polymorphic nucleotide. The backward primer was designed to make the resulting amplicon as short as possible while both primers' Tm values matched with each other for better PCR yield. This short inserted sequence in general is not expected to base pair to the template strand, but rather loops out when the primer is bound to the template. When the complementary strands are copied the inserted sequences are incorporated into the product. Both *Fok*I and *Bst*F5I are typeIIS restriction enzymes that cleave DNA outside the recognition sequence. The *Fok*I enzyme cleaves DNA 9 bases 3' to the recognition site on one strand and 13 bases from the recognition site on the other strand, leaving a four base overhang protruding 5' end. *Bst*F5I cleaves DNA 2 bases 3' to the recognition site on one strand and immediately 3' to the recognition site on the opposite strand, leaving a two base overhang. Thus digestion of the resulting amplification product with *Fok*I followed by *Bst*F5I should result in excision of a 7-mer fragment and a 13-mer fragment. As summarized in Table 1, the 7-mer fragments contain the polymorphic base at the last base of 3'-terminal, and the 13-mer fragments contain the base complementary for the varied site and neighboring four bases. These fragments are then analyzed by mass spectrometry to determine the base identity at the polymorphic site. Inclusion of primer non-encoded 4 nucleotides in addition to the polymorphic site within the 13-mer allows estimation for specific PCR amplification, leading to improved precision in assay interpretation. From the observation that reduction of inserted loop length generally improves assay performance, it is desirable to design the modified primer so that inserted nucleotides are minimized by omitting the redundant sequence if the natural sequence adjacent to the polymorphic site, either 5'-side or the 3'-side, already contains a partial sequence for the restriction endonuclease recognition. The optimal range in the nucleotide length of 5'-arm and 3'-arm sequence across the inserted loop in the engineered primer were found within 16 to 19 and 6 to 8 bases, respectively. Optimal gap for the inserted loop is found to be one base and Tm of the primer pair is desirable to be higher than 65°C. Software for designing the engineered primer and Tm-matched backward primer was developed to integrate the above mentioned empirical measures [see Additional file 1]. The embedding of sequence recognized by two typeIIS restriction enzymes in one primer allows cleavage occurs on both sides of the polymorphic site, releasing fragments that falls in the range to be efficiently analyzed via mass spectrometry without resort to modification of the opposite primer, and universal flexibility in selection of target SNP sites, independently of flanking sequences.

**Table 1:** Primers used for RFMP genotyping assay

SNPs tested	Variation	Sequence of Primer (5'-3')	Polarity
rs1801133	CT	AGCACTTGAAGGAGAAGGTGTggatgTGCGGGAG	Sense
		GCCTCAAAGAAAAGCTGCGTGATG	Antisnese
rs2066462	CT	ACCCAGGCGTCCCCTACCCTGggatgGCTCTCAG	Sense
		GACGTACATCTTCCTCTCGGCGCT	Antisnese
rs1994798	CT	GTGAGGGCCTGCAGACCTTCCggatgTGCAAATA	Sense
		TTCTGCCCTCCCGCTCCCAAGAAC	Antisnese
rs2066470	CT	TTCGAGATGTTCCACCCCGGGggatgCTGGACCC	Sense
		CTCATCTTCTCCCGGAGTCTCTCA	Antisnese
rs1801131	AC	AGATGTGGGGGGAGGAGCTGAggatgCAGTGAAG	Sense
		GTAAAGAACAAAGACTTCAAAGAC	Antisnese

A five-nucleotide sequence (ggatg) embedded in the forward primer to introduce a FokI site in amplicon is indicated by small letters.

RFMP assay established in this study exploits differences in the molecular masses of oligonucleotides comprising the nucleotide variations. The assay is based on amplification and mass detection of oligonucleotides excised from typeIIS enzyme digestion using MALDI-TOF MS. Enzymatic cleavage of the products leads to excision of oligonucleotide fragments representing base variation of the polymorphic site whose mass was determined by MALDI-TOF MS. This genotyping assay represents an improvement over previous methods because it relies on the direct mass determination of PCR products rather than on an indirect analysis, where a fluorescent or radioactive report tag is interpreted. Further, both DNA strands can be analyzed in parallel, and the specific target amplification can be validated simultaneously with mass analysis, providing a level of internal confirmation not achievable by other methods. The use of a typeIIS restriction enzyme makes this assay independent of the fortuitous occurrence of restriction sites, because these enzymes have cleavage sites distal to their recognition sites. Recognition sites are incorporated into the amplification primers, and short fragments that contain the polymorphisms can be generated for mass spectrometric analysis. The RFMP strategy is simple and straightforward, requiring one restriction digestion reaction following target amplification in a single vessel.

### Assay performance and validation

Mass spectra were acquired on a linear MALDI-TOF MS (Biflex IV; Bruker Daltonics) workstation equipped with a 337 nm nitrogen laser and a nominal ion flight path length of 1.25 m as previously described with slight modification [15]. The samples were analyzed in a negative ion mode by using a total acceleration voltage of 20 kV with an 18.25-kV extraction voltage, laser attenuation of 55 and delayed extraction of long time delay. Typically, time-of-flight data from 10 individual laser pulses were recorded and averaged on a transient digitizer with time base of 2 ns and delay of 24000 ns, after which the averaged spectra were automatically converted to mass by accompanying data processing software (Bruker Daltonics Tof 1.6 m). With such settings, the instrument usually provides mass accuracy of 40–80 ppm, mass resolving power of 1500–2000 and sensitivity of 10–50 fmol in the 2–6 kDa mass ranges for oligonucleotides.

**Figure 2:**
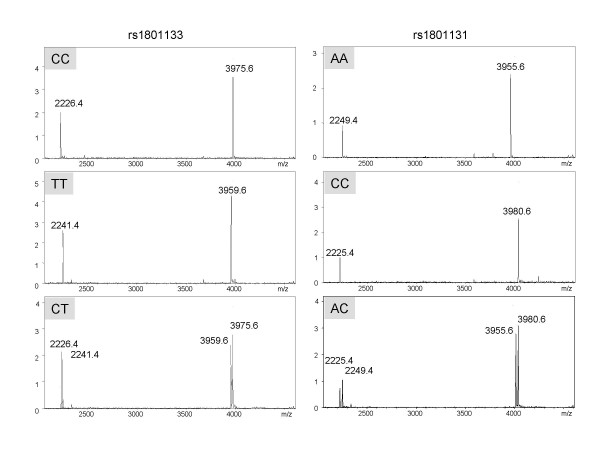
**Representative MALDI-TOF MS spectra in individual samples**. Genotyping results for SNPs rs1801133; masses for a pair of 7 mer and 13 mer for C and T alleles were 2226.4/3975.6 and 2241.4/3959.6, respectivley and rs1801131; masses for a pair of 7 mer and 13 mer for A and C alleles were 2249.4/3955.6 and 2225.4/3980, respectivley. X- and y- axes represent relative ion abundance and mass to charge ratio, respectively.

192 samples were genotyped for 5 SNPs in *MTHFR* gene by RFMP method (Table 2). We successfully called 956 genotypes (99.6%) with an average SNR of 27 for allele-specific signal to non-allele signal (see Figure 2 for representative spectra). Failures were all related with desalting step of restriction enzyme reaction mixtures (0.2%) and spectrum acquisition in MALDI-TOF MS (0.2%). DNA purification, PCR, and restriction enzyme digestion were without failures (Table 3). Accuracy was determined through independent sequencing. Forward and reverse Sanger sequencing were performed and conservative reads were made manually with the identity of the forward and reverse loci blinded at the time of sequence interpretation. Accuracy of Sanger sequencing was measured by comparing reads for which the sequence of both strands existed. 950 of 960 sequence pairs were identical, for an accuracy of 98.9%. Thus the 950 agreeing sequencing pairs were compared to the RFMP genotyping set, giving rise to a concordance rate of 99.8% between both methods (Table 3). The five markers were also genotyped on the identical set of 192 samples by the Snapshot primer extension assay. The assay indicated a concordance rate of 99.5% (945/950) with Sanger sequencing (Table 4). Both the Snapshot and RFMP methods were found to be accurate, robust and required little optimization. Compared for ease of use and throughput considerations, the RFMP assay required less labor for reaction preparations and more advantageous in serial throughput capacity than the Snapshot method (about 8-fold) (Table 4). Time spent on target amplification and post-PCR reactions (single base extension or restriction digestion) was similar, serial throughput was largely dependent on capillary electrophoresis or MS. RFMP produced 18,336 read-outs in a day by automatic data acquisition mode in MS setting while Snapshot called 2,256 genotypes. In terms of cost-effectiveness, we estimated the direct cost per test (reagents and consumables) of the RFMP assay to be about $2 per individual SNP reaction including PCR, restriction digestion, desalting and the running cost including amortization of MS platform (Table 4). The capital equipment costs for the Biflex IV in our laboratory that are estimated to be $50,000 including annual amortization and maintenance are similar to that of automatic sequencer with a 96-capillary system. Though the cost of genotyping per reaction is high dependent on the ability to multiplex reactions and miniaturization of reaction scale, the RFMP assay was estimated to be lower than the Snapshot assay in our hands since slightly higher amortization of MALDI-TOF MS compared to automatic sequencer is quickly offset by its cheaper running cost.

The presence of metal cations produces salt adducts, leading to reduced resolving power and low sensitivity. Various desalting procedures have been established for DNA analyses by MALDI-TOF MS (18–20). We used C18 reverse phase micro-column chromatography as an effective and inexpensive means for desalting oligonucleotides with since it was recyclable by repeated washing with isopropanol up to ten times without remarkable loss of efficiency. We have used fructose as matrix additive for less dependency of the quality of mass spectra on laser power for better ion abundance without compromising mass resolving power as previously described [21,22]. Furthermore, chip-based dispensing on hydrophilic anchors or re-crystallization on matrix-prespotted anchorchip plates allowed robust and controlled, high-density formation of small single crystals.

**Table 2:** Expected masses of oligonucleotides resulting from restriction enzyme cleavage of PCR products for scoring 5 SNPs in *MTHFR* gene by RFMP method

SNPs	Variation	Seuences of restriction fragments^a^		Expected mass (Da)	
		
		7 mer	13 mer	7 mer	13 mer
rs1801133	C	CGGGAGc	ATCGgCTCCCGCA	2226.4	3975.6
	T	CGGGAGt	ATCGaCTCCCGCA	2241.4	3959.6
rs2066462	C	TCTCAGc	GGGCgCTGAGAGC	2136.4	4120.6
	T	TCTCAGt	GGGCaCTGAGAGC	2151.4	4104.6
rs1994798	C	CAAATAc	AGATgTATTTGCA	2153.4	4068.6
	T	CAAATAt	AGATaTATTTGCA	2168.4	4052.6
rs2066470	C	GGACCCc	GCTCgGGGTCCAG	2146.4	4071.6
	T	GGACCCt	GCTCaGGGTCCAG	2161.4	4055.6
rs1801131	A	GTGAAGa	ACTTtCTTCACTG	2249.4	3955.6
	C	GTGAAGc	ACTTgCTTCACTG	2225.4	3980.6

a, the varied bases of the SNP position in oligonucleotide fragments were noted by small letters.

**Table 3:** Performance metrics of RFMP genotyping assay

Measured parameter	Result
Reactions^a^	960
Call rate (%)^b^	956/960 (99.6)
Failures attributable to	
DNA purification	0
PCR	0
Restriction digestion	0
Desalting	2
MALDI-TOF MS	2
Average SNR^b^	27
Repeatability (%)^c^	575/576 (99.8)
Concordance with Sanger Sequencing (%)^d^	948/950 (99.8)

a, total reactions involved determination of the 5 SNP markers in *MTHFR *gene in 192 subjects.

b, average of the ratio of maximum allele signal to maximum non-allele signal of the 5 SNP markers.

c, assessment was based on a new bundle of reactions corresponding to triplicate analysis of 2 SNPs in 96 samples.

d, the comparison includes 4 reactions that failed in the first experimental set-up and were repeated for the attributable steps.

**Table 4:** Comparison of RFMP with Snapshot assays for SNP scoring

	Snapshot	RFMP
Assay chemistry	Minisequencing	Restriction endonuclease digestion
Analyte	Primer extended product	Enzyme-digested amplicon
	(single strand-origin)	(both strands-origin)
Assay platfom	Automatic sequencer	MALDI-TOF MS
Accuracy^a^	99.5%	99.7%
Throughput/day	2,256^b^	18,336
Cost per reaction^c^	US$2.50	US$2.00

a, Concordance with Sanger sequencing

b, With a 96-capillary system

c, The estimations include reagents and consumables for PCR and post PCR reactions, and the running cost and amortization of each detection platform except labor charge.

### Allele frequency determination and haplotype analysis

To test for a relationship between estimated frequencies in pools and direct counts from individuals, ratios were calculated using the mean peak areas generated from multiple mass spectra and compared to the defined pooling ratios. For the markers rs1801133 and rs2066462, we tested if there was a linear relationship between estimated and real allele frequencies. By analysis of artificial pools representing one allele in the frequency range from 0.05 to 0.95 a linear relation of estimated allele frequencies to the expected ratios was observed with the 13 mer fragment (rs1801133: R^2 ^= 0.984, slope = 1.033, p = 0.755; rs2066462: R^2 ^= 0.965, slope = 1.011, p = 0.650). Using the pooling strategy a minor allele with a frequency as low as 0.05 in the pool could be accurately detected for both markers. We have not observed that the significant difference obtained in dynamic range depends on which spectral band is chosen for quantitation between 2 restriction fragments, 7 mer and 13 mer. The 7 mers showed a lower limit of minor allele detection as low as 5%, and a dynamic range of 0.05 to 0.95 with R^2 ^equal to a mean of 0.912 for both markers. Considering that 13 mer fragment has 4 additional bases adjacent to the polymorphism originated from target sequences while the 7 mer has only the polymorphic base, the result suggest better reflection of 13 mer fragment on real abundance due to the advanced target specificity.

**Figure 3:**
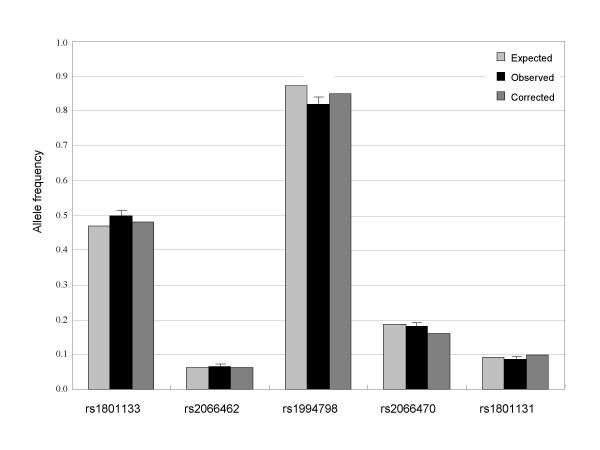
**Allele frequency measurements on pools for different polymorphisms**. Expected allele frequencies are obtained by individual genotyping. The frequencies calculated from pool data were corrected for unequal allelic representation using at least eight mass spectra of heterozygotes. The error bars represent the standard deviation.

Allele frequency estimation of five SNPs selected from public databases was made in pools generated from the Korean population (192 subjects), and compared with results from individual genotyping. The minor allele frequency by the annotation standards of dbSNP database for the particular marker ranged from 0.093 to 0.872 (Fig. 3). The mean of the absolute differences between corrected allele frequency estimates in corresponding pools and the expected frequencies was 0.014 (range from 0.005 to 0.028). To investigate the influence of the pool size on the accuracy and reproducibility of the approach we constructed three pools of different sizes from previously genotyped individuals. Comparing the corrected pool estimates allele frequencies deviated from real frequencies (0.468 for rs1801133) by 0.015, 0.013, and 0.014 in the pools of sizes 96, 144, and 192, respectively. The standard deviations for the corrected pool estimates were 0.018, 0.015, and 0.012. The results suggest that accurate allele frequencies can be determined independently from the number of individuals in the DNA pools tested. The use of MALDI-TOF to determine allele frequencies for pooled samples inevitably lead to limitation in accuracy and sensitivity, considering intrinsic nature of MALDI-TOF measuring relative ion abundance, which is largely influenced by unequal amplification and/or differential ionization efficiency relying on genotype sequences [23,24]. At the current level of precision, the greatest value of the pooling approach is likely to be its suitability as an initial screen to rapidly and cost-efficiently identify which SNPs should undergo individual genotyping within the range of minor allele frequency higher than 0.05 as well as the quantity of DNA used overall. The method required 25 reactions (5 SNPs, and 5 PCR replicates) for initial association testing in contrast to 960 reactions made necessary by individual-sample genotyping for 192 subjects. A follow-up by individual genotyping of selected SNPs showing evidence of association has two major benefits. First, it allows confirmation of the pooled estimates of frequency, and second, it permits the reconstruction of the haplotype showing linkage disequilibrium with the disease.

Hyperhomocysteinemia is caused by low intake of folate and other B vitamins and by genetic factors, including polymorphisms of genes encoding enzymes involved in homocysteine remethylation, such as *MTHFR*, methionine synthase, methionine synthase reductase, and variants of cystathionine synthase, which catalyzes the irreversible step of the transsulfuration pathway [25]. SNPs with documented metabolic and biological effects include *MTHFR *C677T, which is a strong determinant of plasma total homocysteine in individuals with impaired folate status [26]. The polymorphic *MTHFR *mutations (C677T and A1298C) have been suggested as a cause of cardiovascular disease, colorectal neoplasias, neural tube defects, and pregnancy complications, especially in homozygotes for C677T, but also in compound heterozygotes for C677T/A1298C [27,28]. The distribution of 677T and 1298C are known to be worldwide, but those frequencies in different populations vary extensively and the ethnic impact on the association with the clinical phenotypes remains controversial [29].

For analysis of a possible linkage among C677T (rs1801133), A1298C (rs1801131) and other polymorphisms (rs2066470, rs2066462, rs1994798), haplotypes of the five variations in Korean population (N = 192) were built by employing a computational method (EM algorithm). The results showed that the population are composed of 8 haplotypes, and haplotype M1 and M2 were the most prevalent, constituting 71.9% of the samples, which are 677T (rare allele) or 677C (common allele) with residual SNPs all common alleles (Table 5). Of interest, there was a strong association between the 677T allele in exon 4, 31T (common) in intron 6 (rs1994798) and 1298A (common allele) in exon 7. These data suggest that the *MTHFR *677T alteration has occurred once on the 31T-1298A haplotype (93.7%) as previously described by Rosenberg *et al *[27]. It is also noted that 677T allele prefer common alleles in residual 4 loci as shown in haplotype M1, M6, and M8 though M6 and M8 have deviations in rs1994798 and rs2066462, respectivley, suggesting the existence of *MTHFR *677T alteration on a founder haplotype that may have had a selective advantage. The presence of a high prevalence of the specific haplotypes within *MTHFR *gene suggests compounding effects of the SNPs in occurrence of complex diseases and might be worth further investigation for better predicting power in genotype-phenotype association studies.

**Table 5:** Haplotype frequencies inferred from genotype results of 192 Korean subjects determined by RFMP assay

Haplotype	rs2066470 CT (0.188)	rs1801133 CT (0.468)	rs2066462 CT (0.062)	rs1994798 CT (0.872)	rs1801131 AC (0.093)	Counts	Frequency (%)
M1	C	T	C	T	A	144	0.375
M2	C	C	C	T	A	132	0.344
M3	C	C	C	C	C	48	0.125
M4	T	C	T	C	C	28	0.073
M5	T	C	T	C	C	14	0.036
M6	C	T	C	C	A	10	0.026
M7	C	C	T	C	C	4	0.010
M8	C	T	T	T	A	4	0.010
Total						384	1.000

## Conclusion

In conclusion, the RFMP assay for SNP scoring utilizing mass difference of oligonucleotides requires the simple steps of single PCR amplification and restriction enzyme digestion, and is amenable to high-throughput system. The assay represents an improvement over previous methods in reliance on the direct mass determination of PCR products rather than on an indirect analysis, where a base-extended or fluorescent report tag is interpreted, both DNA strands being analyzed in parallel, and the ensured specific target amplification simultaneously with mass analysis, providing an additional level of assay precision. Using RFMP assay, we demonstrated highly reliable genotyping of five SNP markers in *MTHFR *gene, known to be associated with hyperhomocysteinemia and cardiovascular diseases, and also provided the potential for application to determination of allele frequencies in DNA pools as a means of efficiently screening SNPs and prioritizing them for further study. Therefore, we believe that the simplicity, accuracy and amenability to high-throughput screening analysis make the RFMP assay suitable for large-scale genotype association study and a routine genotyping platform in clinical laboratories.

## Methods

### SNP markers and primer design

SNPs rs1801133, rs2066462, rs1994798, rs2066470, and rs1801131 (see Table 1) were selected from the NCBI dbSNP database [30] and their flanking sequences were retrieved from the UCSC genome browser [31]. Primers were designed using proprietary software (PickCamp ver9, GeneMatrix) and synthesized by Bioneer Ltd. (Seoul, Korea). PickCamp software beta-version is available as Additional file 1 of this article.

### PCR amplification

Informed consent was obtained form all subjects and experimental protocol conformed to the ethical guidelines of the 1975 Declaration of Helsinki. 50 ng of the genomic DNA was used for the PCR reaction. PCR was performed in 18 μl reaction mixture containing 20 mM Tris-HCl (pH 8.4), 50 mM KCl, 0.2 mM of each dNTP, 10 pmol of each primer, and 0.4 units of Platinum^® ^*Taq *DNA polymerase (Invitrogen, Carlsbad, CA). The amplification conditions included initial denaturation at 94°C for 2 min, 10 cycles of denaturation at 94°C for 15 sec, annealing at 50°C for 15 sec and extension at 72°C for 30 sec, followed by 35 cycles of denaturation at 94°C for 15 sec, annealing at 55°C for 15 sec, and extension at 72°C for 30 sec. The respective sequences of forward and reverse primers used in the PCR for each SNP site are summarized in Table 1. Sequences underlined in each primer were engineered to insert new *Fok*I site in amplicon as shown in Figure 1.

### Restriction enzyme digestion, desalting and MALDI-TOF analysis

Restriction enzyme digestion of PCR products was performed by mixing the PCR reaction mixture with 10 μl of buffer containing 50 mM potassium acetate, 20 mM Tris-acetate, 10 mM magnesium acetate, 1 mM dithiothreitol, and 1 unit of each *Fok*I and *Bst*F5I at 37°C for 15 min. The resulting digest was purified by vacuum filtration through a 384-well sample preparation plate containing 5 mg of polymeric sorbent (Waters, Milford, MA) per well using Microlab 4200 robotic liquid handler (Hamilton, Reno, NV). Each well was equilibrated with 90 μl of 1 M triethylammoninumacetate (pH 7.6). Each cleavage reaction mixture was added to 70 μl of 1 M TEAA, pH 7.6 and loaded into a well. After rinsing 5 times with 85 μl of 0.1 M TEAA pH 7.0, the plate was reassembled on a vacuum manifold and eluted with 60 μl of 60% aqueous isopropanol into a collection plate, which was placed on a heating block at 115°C for 90 min. The desalted reaction mixtures were resuspended with matrix solution containing 50 mg/ml 3-hydroxy picolinic acid, 0.05 M ammonium citrate, 5 mg/ml of fructose, and 30% acetonitrile, and were spotted in 3 μl volumes on a polished anchorchip plate (Bruker Daltonics, Billerica, MA) using Microlab 4200 robotics or resuspended with distilled water and dotted in 2 μl to pre-spotted anchorchip plate which had only matrix crystallized in advance. Mass spectra were acquired on a linear MALDI-TOF MS (Bruker Daltonics Biflex IV) workstation. Spectra were acquired in a positive ion, delayed extraction mode. Typically, time-of-flight data from 20 – 50 individual laser pulses were recorded and averaged on a transient digitizer, after which the averaged spectra were automatically converted to mass by data processing software (Bruker Daltonics Genotools version 1.0).

### DNA sequencing and Snapshot assay

The RFMP results were compared with the results from either direct sequencing or the clonal sequencing assay. When direct sequencing results were not decisive, we cloned the PCR products into the pCR-Script Amp cloning vector (Stratagene, La Jolla, CA), for sequence analysis of each clone. Sequence analysis was performed by ABI PRISM 310 Genetic Analyzer (Applied Biosystems, New York, NY). The primers used for Snapshot assay were designed and the primer extension reactions were carried out with SnaPshot™ multiplex mix (Applied Biosystems) according to manufacturer's recommendations. The reaction mixtures were run on ABI3700 (Applied Biosystems) using POP6 polymer and analyzed by Genescan program.

### Determination of allele frequencies in DNA pools

The concentration of the DNAs used to construct pools was measured using the Picogreen reagents and kits (Molecular Probes, Eugene, OR). The DNAs were diluted to a final concentration of 8 ng/μl and equal amounts of DNA were mixed to form the pools. Range pools were constructed by mixing appropriate volumes of homozygote DNA. The concentrations ranged from 50–50% to 95-5%, with 5% increments. Allele frequencies were calculated using peak areas generated from mass spectra. All spectra were smoothed by applying a 21-point Savitzky-Golay filter function (Bruker Daltonics XMASS) to minimize noise errors. Peak areas were estimated using TOF 1.6 m taking into account baseline correction and the noise level of the spectrum. To evaluate the reproducibility of the frequency estimates, assays were performed in 5 replicates for each pool and marker. In order to take unequal representation of both alleles in the mass spectrum into account, at least five heterozygotes were genotyped individually as recommended by Le Hellard *et al *[24]. The mean of the ratios obtained from the peak areas was used to correct the final allele frequency estimates [29].

## Abbreviations

MALDI-TOF: matrix-assisted laser desorption ionization time-of-flight; RFMP: restriction fragment mass polymorphism; PCR: polymerase chain reaction; MS: mass spectrometry; RFLP: restriction fragment length polymorphism; SNPs: single nucleotide polymorphisms; MTHFR: methylenetetrahydrofolate reductase.

## Authors' contributions

Experiments were designed by SPH, WY and S–OK. SPH, SIJ, SKS and SYH performed the experiments. SPH, WY, H–BO, SHL, SDL and S–OK analyzed the data. HR contributed software and analysis tools. SPH and S–OK wrote the paper. All authors read and approved the final manuscript.

## Supplementary Material

Additional file 1 PickCamp ver9. Software for designing engineered primers and calculating predicted mass values for SNP scoring by RFMP assay. Click here for file
